# The complete mitochondrial DNA of *Gymnocypris potanini firmispinatus* and comparative mitogenomic analyses of the *Gymnocypris* species

**DOI:** 10.1080/23802359.2016.1180556

**Published:** 2016-07-07

**Authors:** Jiaxiang Hu, Yueping Cai, Sen Xiong, Zhide He, Song Li, Xiongyan Wang, Zhi He, Taiming Yan

**Affiliations:** College of Animal Science and Technology, Sichuan Agricultural University, Chengdu, China

**Keywords:** *Gymnocypris potanini firmispinatus*, mitochondrial genome, phylogenetic analyses, taxonomic status

## Abstract

The complete mitochondrial genome of *Gymnocypris potanini firmispinatus* was sequenced and compared with others *Gymnocypris* species. The mitochondrial genome, consisting of 16,680 base pairs (bp), encoded 13 protein-coding genes, 2 ribosomal RNAs, 22 transfer RNAs and a non-coding control region, as those found in other *Gymnocypris* species. These results can provide useful data for further studies on taxonomic status, molecular systematics and stock evaluation.

*Gymnocypris potanini firmispinatus*, a subspecies of *G. potanini*, is only distributed in Jinsha River, the mainstream of Yangtze River, and Yongchun River, a tributary of Lancang River (Yue et al. 2000). In recently years, activities like overfishing, dam construction, water pollution and other human interference may exert high pressure on fisheries. Thus, to benefit the effective management of *G. potanini firmispinatus* resource, some basic biology data including genetic information should be further studied. The mitogenome sequence information may be useful in systematics, resource protection and development.

In the present study, one specimen *G. potanini firmispinatus* chosen for mitochondrial genome analysis were collected from the upstream of Jinsha River (N′28°04′29.71″, E′102°25′57.90″) (Specimen is stored in Aquaculture Department of Sichuan Agricultural University, number gp20130102). Primers were designed for polymerase chain reaction (PCR) amplification and sequencing on the basis of the mitogenome sequence of *G. eckloni* Herzensten (GenBank Accession No. JQ0042791) (Qi et al. 2013). The complete mt genome of *G. potanini firmispinatus* was 16,680 bp in length and has been deposited in GenBank with Accession No. KU896808. The mitochondrial genome encoded 13 protein-coding genes, 2 ribosomal RNAs, 22 transfer RNAs and a non-coding control region, as those found in other *Gymnocypris* species (Qi et al. 2013; Qiao et al. 2014; Zhang et al. 2016). The nucleotide composition of the genome of *G. potanini firmispinatus* is A 28.4%, T 27.2%, G 18.4%, and C 26.0%, with a low A + T content of 56.6%. Except for the nad6 and eight tRNA genes (tRNA-Gln, tRNA-Aln, tRNA-Asn, tRNA-Lys, tRNA-Tyr, tRNA-SerUCN, tRNA-Glu, and tRNA-Pro) encoded on the light-strand, all others genes were encoded on the heavy-strand. This is a typical gene arrangement conforming to the other Gymnocypris species and vertebrate consensus (Qi et al. 2013; Qiao et al. 2014).

All genes use ATG as a start codon, except *cox1* use GTG, which was also discovered in other *Gymnocypris* species (Qi et al. 2013; Qiao et al. 2014). Most open reading frames ended with two types of complete stop codons TAA and TAG, whereas few genes (including cox2, nd4 and cob) had an incomplete stop codon: T––. These results showed that the PCGs are stable among the *Gymnocypris* species.

Based on combined nucleotide sequence data of 12 heavy-strand protein-coding genes of *G. potanini firmispinatus*, and together with the sequences of other *Gymnocypris* fishes, phylogenetic trees were constructed using the ME methods (Figure. 1). All *Gymnocypris* species had close relationship, *G. przewalskii*, *G. przewalskii ganzihonensis*, and *G. eckloni* were monophyletic in the trees, which were consistent based on the mitochondrial DNA cytochrome b gene or mitogenoma sequences. In agreement with the findings of previous molecular analyses (Qi et al. 2013; Zhang et al. 2015), *G. dobula* and *G. namensis* also had a close genetic relationship, however, which had a closer distance with *G. eckloni chilianensis* as the subspecies of *G. eckloni* (Zhang et al. 2015). Thus, the mitochondrial genome data and phylogenetic analysis of the *G. potanini firmispinatus* can enrich the evolution research of Gymnocypris.

**Figure 1.**
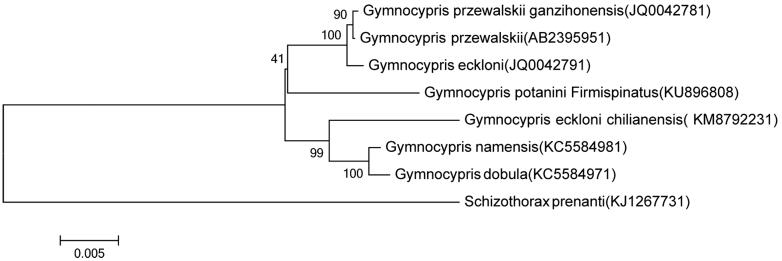
Phylogenetic relationships among 7 species of *Gymnocypris* inferred from minimum evolution of deduced amino acid sequences of 12 mitochondrial proteins. The numbers on the branches are bootstrap values for ME.
